# Stroke after Initiating IV Penicillin for Neurosyphilis: A Possible Jarisch-Herxheimer Reaction

**DOI:** 10.1155/2014/548179

**Published:** 2014-11-05

**Authors:** Vineet Punia, Appaji Rayi, Adithya Sivaraju

**Affiliations:** ^1^Epilepsy Centre, Neurological Institute, Cleveland Clinic, 9500 Euclid Avenue, Cleveland, OH 44106, USA; ^2^Department of Neurology, SUNY Downstate Medical Center, 450 Clarkson Avenue, P.O. Box 1275, Brooklyn, NY 11203, USA; ^3^Department of Neurology, Epilepsy Section, Yale University School of Medicine, 800 Howard Avenue, New Haven, CT 06510, USA

## Abstract

*Introduction.* Syphilis incidence has increased in the US in the last decade. Jarisch-Herxheimer reaction (JHR) is a well-documented adverse effect of penicillin treatment in syphilis. Stroke has not been reported as part of its phenomenology. *Case Report.* A 57-year-old man presented with worsening memory. His minimental status examination score was 14/30. Serum RPR test was positive and VDRL test in the CSF was reactive. Within six hours of first dose of IV crystalline penicillin G, he was found to have hemineglect and difficulty moving the left leg. MRI of the brain showed multiple acute ischemic strokes. Immediate MRA ruled out vascular occlusion. Penicillin treatment was stopped. Four hours later, he was found to be febrile and had two episodes of generalized tonic-clonic seizures. *Conclusions.* We report a case of confirmed neurosyphilis with no known modifiable stroke risk factors, who developed acute ischemic stroke and other constitutional symptoms consistent with JHR after IV penicillin. This is the first reported case in literature where an acute ischemic stroke can be attributed to Jarisch-Herxheimer reaction. Given an increase in incidence of syphilis in recent years, our case underlies the importance of keeping in mind potential catastrophic drug adverse reactions in neurosyphilis patients.

## 1. Introduction

Infectious diseases are relatively rare cause of stroke, but they may be a significant contributor in patients who lack conventional risk factors. Syphilis is one such infection. Syphilis patient may suffer stroke as its only clinical manifestation during early and secondary forms [1]. Early neurosyphilis, more specifically syphilitic meningovasculitis, often presents with stroke [2]. Neurosyphilis was quite common in the preantibiotic era, but its incidence dropped dramatically with the introduction of penicillin, which is still the mainstay of therapy.

Penicillin therapy in syphilis, like in many other spirochetal infections, can potentially cause Jarisch-Herxheimer reaction (JHR). Symptomatic JHR has been reported in 1.7 to 11% of neurosyphilis patients [3]. It is typically characterized by fever, chills, myalgia, headache, hyperventilation, hemodynamic instability, leukocytosis, and exacerbation of skin lesions [4]. In patients with dementia paralytica, it is also associated with seizures, exacerbation of preexisting psychosis, or the development of focal neurological deficits [5]. It typically starts within 1-2 hours of the start of penicillin therapy but delayed onset up to 12–24 hours in patients with late neurosyphilis has been reported in literature [6].

There is only one report of stroke after penicillin treatment initiation in a patient with neurosyphilis [3]. But the authors proposed JHR to be a less likely causative factor for stroke in that patient. In the current report, we present the case of a patient who developed strokes in multiple vascular territories within hours of initiation of IV penicillin therapy for treatment of confirmed neurosyphilis. We propose that JHR is the most likely cause of stroke in this patient.

## 2. Case Presentation

**Figure 1:**
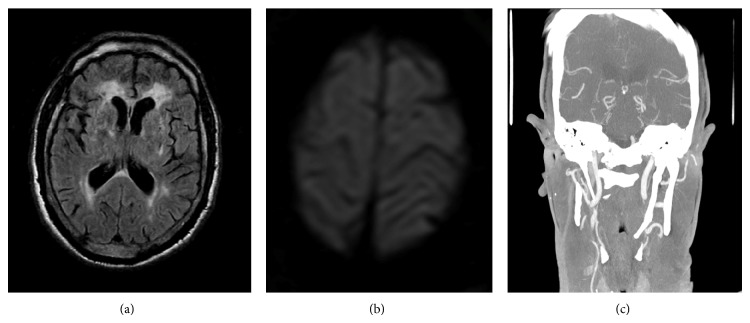
Pre-penicillin therapy imaging. (a) FLAIR, (b) DWI sequence, and (c) CT angiogram.

**Figure 2:**
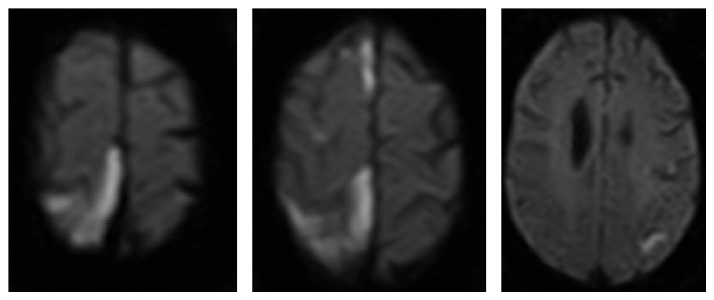
Diffusion sequence immediately after symptom onset.

A 57-year-old African American man with a history of alcohol abuse, in remission for several years, was brought to the ER due to worsening memory. Patient was a very poor historian and could not provide any pertinent information. On examination, his vitals were within normal limits. He had a flat affect and scored 14/30 on minimental status examination (MMSE). His cranial nerves examination showed no deficits. His strength was 5/5 in all muscle groups except for 4+/5 in left iliopsoas. Sensory examination showed mildly reduced sensation for vibration and pinprick in bilateral feet. He had Babinski reflex present in the left foot, positive Romberg sign, and walked with a wide base. Urine toxicology was negative, alcohol level <10 mg/dL, HbA1C 5.4%, LDL 64 mg/dL, TSH 2.07 mcUI/mL, vitamin B12 402 pg/mL, folate 13.6 ng/mL, and HIV screen negative. MRI of the brain (Figure 1(a)) showed moderate leukoaraiosis and global volume loss. CTA (Figure 1(c)) showed right dominant vertebral artery, mild calcification of the cavernous segment of the left ICA without stenosis, and a hypoplastic A1 segment of the right ACA. Transthoracic echo showed an EF of 50% and no PFO or intracardiac shunt by bubble study or any other valvular abnormalities. His serum RPR was reactive (1 : 32 titer) along with a reactive MHA-TP. Patient had negative antiphospholipid antibody. Patient got one dose of benzathine penicillin 2.4 million units IM for latent syphilis. A lumbar puncture CSF showed glucose of 69 mg/dL, protein 62.4 mg/dL, RBC 5/cmm, and WBC 3/cmm (95% lymphocytes, 5% monocytes) in CSF. Gram stain and CSF culture were negative. CSF VDRL was reactive with a titer of 1 : 8 and he was started on IV aqueous crystalline penicillin G 3 million units q4 hourly for confirmed neurosyphilis. Six hours after the first dose, patient was found on floor. His vitals were stable on examination. His mental status was grossly unchanged but he had difficulty moving his left leg and had neglect for the left half of his body along with new onset mild dysarthria. Immediate MRI showed infarcts as shown in Figure 2. A MRA done at the same time showed findings similar to recent CTA. He was transferred to a telemonitored bed. Four hours later, patient was seen to have profuse sweating with spike in temperature of 100.6, HR 130, RR 40, and BP 190/110. Soon, he had 2 episodes of GTC seizures lasting for 2 minutes each. Labs sent at the time showed a transient leukocytosis of 14.4. Body temperature normalized over the next 4 hours. Cardiac rhythm during the course of his stay in telemonitored bed did not show any abnormality. Penicillin was discontinued temporarily and was restarted next day at 2 million units q4 hour IV. Patient did not receive steroids prior to treatment initiation.

## 3. Discussion

Syphilis has made a resurgence after the HIV epidemic and its incidence has been increasing since beginning of this millennium [7, 8]. Our patient had confirmed neurosyphilis based on positive CSF VDRL. His clinical picture resembled dementia paralytica stage of neurosyphilis. It may also explain the lack of pleocytosis in CSF as 10% of patients with late “burned out” stage of neurosyphilis lack pleocytosis [8]. Our patient lacked any conventional stroke risk factors. Patients with meningovascular stage of neurosyphilis develop transmural proliferative arteritis, which can involve large-medium vessels (Heubner's artery), especially middle cerebral artery or small perforating end arteries (Nissl's arteritis) [9, 10] causing stroke. The benign CSF picture, unremarkable vessel imaging, and his initial clinical presentation rule out meningovascular stage of neurosyphilis in our patient.

JHR has been reported to occur in up to 75% of cases of dementia paralytica [11]. At a histological level, JHR causes congestion in vessels along with swelling of endothelial cells [5]. JHR was not seen after benzathine penicillin G in our patient because it does not cross blood-brain barrier to reach treponemicidal levels [8]. We do not have pathological evidence to implicate JHR as the cause of stroke in our patient, but the facts that back our hypothesis are as follows:

- lack of other conventional stroke risk factors (including normal echocardiogram and heart rhythm monitoring during telemonitoring),
- unlikelihood of patient having meningovascular neurosyphilis (as explained above),
- temporal relation of IV aqueous crystalline penicillin G with onset of stroke,
- typical JHR reaction features along with seizures (typical of JHR seen in such patient population [5]) around the time of stroke.

However, other possibilities cannot be ruled out. The temporal relation could be just a coincidence. Also the infarct in the right parietal lobe seems to be caused by involvement of a medium to large vessel, which is less likely to be occluded by JHR. TNF-*α* and IL-6 play a major role in the pathogenesis of JHR with evidence of sudden peak in their levels at the onset of JHR [12]. These cytokines mediate the ischemic pathogenesis in brain infarct [13] and their overproduction during JHR may cause infarction size to be much more than expected just from vessel occlusion.

There are reports of JHR causing worsening or new onset symptoms like Bell's palsy [14], status epilepticus [15], optic neuropathy [16], worsened MRI findings, and behavioral issues [17]. But there is no report in literature to the best of author's knowledge reporting ischemic strokes secondary to JHR. There is only one other case in literature that invokes JHR as a possible cause of stroke. It reports on a patient who developed stroke four days after penicillin. The authors doubted JHR as a cause of the stroke because of lack of fever (a cardinal JHR feature) [3]. In our opinion, a delay of four days from initiation of penicillin therapy also make JHR an unlikely cause of stroke in the reported case. Nonetheless, the authors concluded that JHR “may have played a role.” But, in our patient, the temporal correlate of events after first penicillin G and the symptom spectrum were very typical of JHR.

In conclusion, JHR is a clinical entity that needs wider recognition and should be considered prior to IV penicillin initiation in patients with neurosyphilis.
